# Unwinding the Neurovascular Unit: The Vascular Extracellular Matrix and Mechanochemical Collagen Functionalization in Vascular Dementia

**DOI:** 10.3390/ijms27146521

**Published:** 2026-07-22

**Authors:** Annah Ellingson, Aruna Kalyanasundaram, Joseph P. R. O. Orgel

**Affiliations:** 1 Department of Biology, Illinois Institute of Technology, Chicago, IL 60616, USA; 2 The Center for Molecular Study of Condensed Soft Matter, Illinois Institute of Technology, Chicago, IL 60616, USA; 3 Department of Biomedical Engineering, Illinois Institute of Technology, Chicago, IL 60616, USA

**Keywords:** extracellular matrix, vascular dementia, collagen, matrix metalloproteinases, mechanotransduction, blood–brain barrier, neurovascular unit

## Abstract

Vascular dementia (VaD) represents a spectrum of neurodegenerative disorders driven by chronic or acute cerebral hypoperfusion, converging on the structural collapse of the neurovascular unit (NVU). While vascular mechanics and neuroinflammation have been extensively characterized, the extracellular matrix (ECM) remains an under-appreciated driver of this pathology. Far from being a passive scaffold, the ECM serves as a dynamic regulator of brain homeostasis, governing the integrity of the blood–brain barrier (BBB), supporting synaptic plasticity via Perineuronal Nets (PNNs), and facilitating the glymphatic clearance of metabolic waste. This review and perspective examines the pivotal role of the ECM within the “tripartite” NVU, the interface connecting vascular cells, CNS glia, and perivascular nerves. We discuss how the dysregulated activity of matrix metalloproteinases (MMPs) and the alteration of basement membrane components compromise the architectural defenses of the NVU. We describe how these structural failures can transform vascular insults into progressive neurodegenerative decline by unmasking inflammatory binding sites and disrupting cell–matrix signaling. Specifically, we highlight (1) the physiological architecture of the NVU and its dependence on specific collagen organization, (2) the mechanistic pathways of ECM disruption in VaD pathogenesis, including the feedback loop between ischemia and proteolysis, (3) the emerging therapeutic potential of targeting matrix components to restore neurovascular stability, and (4) a novel mechanochemical hypothesis framing pathological collagen functionalization as an epidemiological bimodal switch. Uncovering the interplay between structural remodeling and vascular function offers novel targets to halt the progression of dementia.

## 1. Foundations of the Neurovascular Unit and Clinical Criteria

### 1.1. Introduction

Vascular dementia (VaD) is the second most common form of dementia, representing a heterogeneous spectrum of neurodegenerative disorders united by a common etiology: the chronic or acute insufficiency of blood supply to neuronal tissues [1,2]. Whether driven by catastrophic events such as stroke and hemorrhage, or the insidious progression of arteriosclerosis, hypertension, and small vessel disease, these pathologies converge on a shared endpoint—progressive cognitive decline driven by neurovascular unit (NVU) dysfunction [3,4]. While ischemia elicits an endogenous inflammatory response intended to aid neural plasticity and remodeling, in VaD, this response becomes maladaptive. The resulting dysregulated inflammation drives synaptic loss and, critically, the overactivation of the metalloproteinase system [5].

However, while considerable attention has been focused on vascular mechanics and neuroinflammation, there remains a significant gap in knowledge regarding the role of the ECM as a dynamic regulator of this pathology [6]. Far from being a passive scaffold, the ECM is an active participant in brain health: it maintains the physical integrity of the BBB, supports synaptic function and plasticity, and facilitates the glymphatic clearance of metabolic waste [7,8]. Its degradation disrupts cell–matrix homeostasis and promotes fibrosis, a pathogenic cycle that exacerbates hypoperfusion and atrophy [6].

### 1.2. The Neurovascular Unit and ECM Architecture

**Figure 1:**
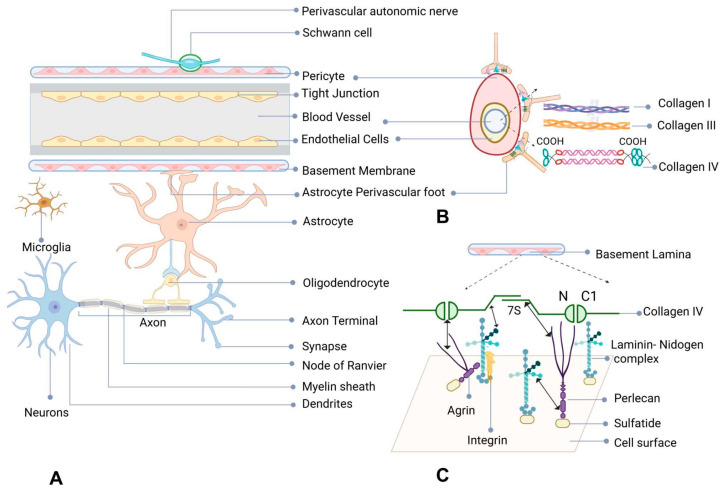
The architecture of the neurovascular unit (NVU). (**A**) Schematic representation of the NVU: Illustrates the functional coupling between the vasculature and neuronal tissues. The astrocytic end-feet form a critical bridge, connecting to the perivascular cells within the basement lamina of the blood vessel and extending to interact with neurons and oligodendrocytes. This structural integration facilitates signaling, metabolic support, and repair. Note that while the central neuronal axons are myelinated by oligodendrocytes, the perivascular autonomic fibers are ensheathed by Schwann cells, reflecting the CNS-PNS transition at the vascular interface. (**B**) Cross-section of the NVU extracellular matrix: Fibrillar collagens, specifically Type I and Type III, provide the necessary tensile strength and structural rigidity to the vessel wall. The ratio of these collagen types is critical for maintaining vascular compliance and supporting cellular repair mechanisms. (**C**) Molecular organization of the basement membrane (basal lamina): This specialized matrix is defined by a network of Type IV collagen, which interacts with laminin, nidogen, and heparan sulfate proteoglycans (perlecan and agrin). The laminin and collagen networks are cross-linked by the laminin–nidogen complex (double arrow) and tethered to the cell surface via integrins and sulfated glycolipids (sulfatides), creating a selective barrier essential for BBB integrity. Created in BioRender. Kalyanasundaram, A. (2026) https://BioRender.com/mduxb0y.

This review examines the NVU not merely as a cellular interface, but as a complex architectural assembly (Figure 1). Functionally, the NVU acts as a cohesive module comprising endothelial cells, pericytes, astrocytes, microglia, and neurons [7,8]. Crucially, however, this review considers an extended framework wherein this module functions as a “tripartite” junction connecting CNS glia, vascular cells, and the extrinsic perivascular nerves of the peripheral nervous system (PNS). While incorporating perivascular nerves extends the conventional definition of the NVU, this juxtaposition highlights a unique functional transition zone. While central axons in the parenchyma are myelinated by oligodendrocytes, the blood vessels themselves are often innervated by sympathetic and sensory fibers ensheathed by Schwann cells. This interface between CNS and PNS elements renders the NVU particularly susceptible to complex signaling failures during vascular pathology.

Within this unit, the ECM provides the essential structural cues that maintain cohesion across these distinct compartments. Astrocytic end-feet, which bridge the vasculature and neuronal circuitry, interact directly with the basement lamina to regulate signaling and metabolic coupling [3]. In VaD, the disruption of this specific architecture, mediated by MMPs and altered proteoglycan expression, precipitates the failure of the BBB and the onset of neuroinflammation. We herein detail how the structural compromise of the ECM drives VaD progression and highlight emerging therapeutic targets to restore neurovascular stability.

### 1.3. Criteria to Define Vascular Dementia

**Figure 2:**
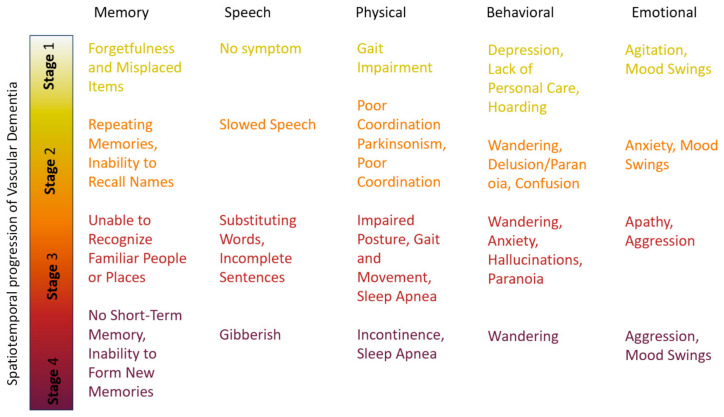
Proposed conceptual framework for the spatiotemporal progression of NVU pathology (see Table 1). The disease is classified into 4 stages: Stage 1 (light yellow) represents the cognitive impairment, Stage 2 (dark yellow) exhibits traits of early-stage dementia, Stage 3 (orange) refers to middle-stage dementia and Stage 4 (red) indicates late-stage dementia. For each stage the illustration lists the physical attribute in terms of the memory, speech, physical, behavioral and Emotional aspects. The color scheme moves from yellow to red with increasing severity; the continuous color gradient is intended to indicate that these are not distinct, isolated phases, but rather a fluid continuum of increasing severity.

VaD has a heterogeneous nature. With no singular test, accurate diagnosis and identification of appropriate courses of treatment have required the development of defined criteria. Scheltens and Hijdra [9] reviewed various such criteria, and based upon their analysis, they concluded that the state of California Alzheimer’s Disease Diagnostic and Treatment Centers (ADDTC) and the National Institute of Neurological Disorders and Stroke and the Association Internationale pour la Recherche et l’Enseignement en Neurosciences (NINDS-AIREN) were the only ones of clinical utility [10,11]. The various scales that have been developed are generally based upon a stroke-like presentation, with resultant cognitive impairment temporally related to the ictus, along with supportive neuroimaging findings and risk factors for stroke. VaD is characterized by problems with memory, judgment, executive function, and an overall decline in cognitive ability with the NVU being highlighted as the underlying therapeutic target [12]. To synthesize how these highly heterogeneous vascular insults translate into chronic neurodegenerative decline over time, we present a four-tier conceptual framework to outline the spatiotemporal progression of NVU pathology (Table 1). Rather than a rigid timeline or clinical diagnostic scale mirroring the linear progression of Alzheimer’s disease, this reflects how micro-mechanical and molecular matrix alterations accumulate and, alongside Table 1, Table 2 and Table 3 and Figure 2, helps form a conceptual bridge to identify molecular mechanisms.

## 2. Extracellular Matrix Vulnerability and Ischemic Pathology

### 2.1. How Vascular Dementia Impacts the ECM of the Brain

The extracellular matrix (ECM) constitutes 10–20% of total brain volume, providing structural support and mediating cell–cell interactions [7,14]. It plays crucial roles in development, aging, and neurodegeneration, particularly at the NVU and blood–brain barrier [8]. The ECM composition is dynamic and region-specific, consisting of over one thousand proteins, including collagens, laminins, fibronectin, and nidogens [15]. ECM components form diffuse matrices, PNNs, perisynaptic nets, and basement membranes [7,16]. The ECM regulates cell migration, axonal guidance, synaptogenesis, and repair following injury [7,14,16]. Changes in ECM composition are associated with cerebrovascular diseases, with core matrisome genes like COL4A1, COL4A2, VCAN, and APOE being significantly enriched in the cerebral ECM network [15]. Modulating ECM composition may be a potential therapeutic target for traumatic brain injury and other neurological disorders [14].

As alluded to already, VaD is not a single disease; it encompasses a broad spectrum of subtypes. Table 2 contains a broad classification of subtypes and provides an overview of the changes in ECM components for the different types of VaD. Each affects the neuronal ECM in a unique fashion.

**Table 2:** Overlapping and convergent alterations in the neurovascular extracellular matrix across distinct clinical phenotypes of vascular dementia.

VaD Type	Description	Impact on ECM	Affected ECM Components	Key Biological Proteins and Components	References
Single-Infarct Dementia	Caused by a single large stroke in a non-redundant brain region; differs from multi-infarct dementia by its single lesion origin.	Localized ECM damage at infarct site	Matrix disruption; BBB weakening	MMP-9, laminin, fibronectin, collagen IV	[5,17]
Strategic Infarct Dementia	Results from infarcts in specific areas critical for cognition (e.g., thalamus, angular gyrus); differs by precise localization and abrupt symptom onset.	Targeted ECM degradation in key cognitive areas	Regional proteoglycan and glycoprotein loss	Fibronectin, laminin, tenascin, thrombospondin	[11,15]
Subcortical Ischemic VaD (SIVD/Binswanger’s Disease)	Caused by small vessel disease and chronic white matter ischemia. Distinct from MID by diffuse rather than focal infarcts.	Chronic ischemia leads to ECM breakdown in white matter	Demyelination, basement membrane thickening	MMP-2/TIMP-3 ratio, MMP-9, ADAMTS-4/5, agrin, perlecan, nidogen, collagen IV	[18,19]
Multi-Infarct Dementia	Arises from multiple strokes over time; unlike SIVD, involves larger and more varied infarct zones including cortical regions.	Repeated infarcts cause cumulative ECM damage	Diffuse ECM degradation; astrocytic gliosis	GFAP, tenascin-C, MMP-3, MMP-9/TIMP-1, collagen I and IV	[11,17]
Mixed Dementia (VaD + Alzheimer’s)	Involves overlapping pathologies of VaD and AD; combines vascular lesions with amyloid plaques and tau tangles.	Synergistic ECM disruption and amyloid deposition	ECM binds amyloid; increases stiffness, impairs clearance	Amyloid-β, HSPGs (agrin, perlecan), MMP-9, collagen IV matricryptins, ADAMTS	[18,20,21,22]
Post-Stroke Dementia	Develops after a major clinical stroke; may occur immediately or months later; differs from MID by temporal link to a single stroke event.	Acute ECM remodeling; inflammatory response	ECM-cleaving enzymes activated; edema	MMP-2, MMP-9, ADAMTS, laminin, versican, brevican	[18]
Hereditary VaD (CADASIL/CARASIL)	Caused by genetic mutations (e.g., NOTCH3, HTRA1); early-onset and progressive; differs by familial inheritance and known molecular drivers.	Genetic ECM protein accumulation in vessels	ECM stiffening and occlusion	NOTCH3 (CADASIL), HTRA1 (CARASIL), collagen IV, TIMPs	[11,15]
Hypoperfusion VaD	Caused by chronic global reduction in cerebral perfusion (e.g., heart failure); distinct from infarct-related VaD by diffuse and non-focal pattern.	Global ECM stress due to reduced perfusion	ECM loss in watershed areas; BBB vulnerability	MMP-2, perlecan, fibronectin, biglycan, decorin	[17]

### 2.2. Pathology of Vascular Dementia

The broad spectrum of neurodegenerative conditions are linked in etiology by failures or interruptions to the blood supply to the brain (Figure 2). Disruptions in blood supply lead to tissue damage and cell death, a process known as infarction, which can happen either by blockages or bleeds. This condition can onset either progressively or acutely depending on the nature of the infarction [1,2]. Small blockages and bleeds over time can result in a slow onset of the disease while a single stroke or a traumatic brain injury may result in a more acute onset following the event. VaD is facilitated by conditions where the blood vessels are hardened or constricted. Hypertension, arterial plaque-buildup and aging give rise to these changes in the vasculature that result in a chronic inability for the blood vessels to accommodate neural oxygen demand, setting the stage for VaD pathogenesis. When blood vessels undergo excessive hardening, their ability to supply the correct amount of oxygen to a particular tissue is impeded as oxygen demand fluctuates with neural activity [4]. Due to the anatomy of the vasculature, sclerotic vessels in the brain, particularly in the hippocampus, are especially vulnerable to creating ischemic conditions that lead to infarcts [3].

**Figure 3:**
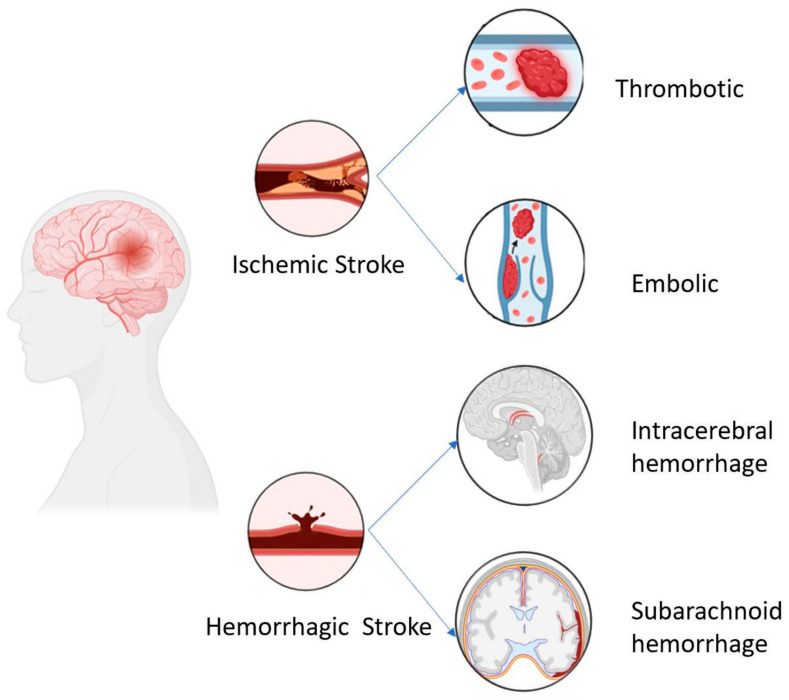
Mechanistic cause of Vascular Dementia is reduction or blockage of blood flow to parts of the brain. Adapted and reproduced from [25] under the terms of the Creative Commons Attribution License (CC BY).

Sclerosis also causes mechanical stress on the vessels as they cannot accommodate higher blood volumes, thereby damaging and weakening them. This may result in leaks ranging from microbleeds to severe hemorrhages which also further deprive neuronal tissue of oxygen [23]. Buildup of plaque in the blood vessels, or atherosclerosis, also contributes to ischemia in the brain. This accumulation of plaque not only promotes the hardening of blood vessels, but can also lead to physical interruptions of blood flow through vessels. The classification of hemorrhagic and ischemic conditions is shown in Figure 3. Vascular disease also increases the risk of blood clots which can obstruct vessels in the brain. These blockages may induce a range of infarcts, from microinfarcts to significant strokes, both of which are prominent risk factors of VaD. In fact, the more strokes a person suffers, the greater the risk of developing VaD, or multi-infarct dementia in this case [24].

Stroke is a major cause of VD. Stroke can be broadly classified as Ischemic stroke, in which there is a reduction in the blood flow to the brain due to blockage in the blood vessel, and Hemorrhagic Stroke where the brain blood vessels break and bleed. Ischemic stroke is further divided into two subdivisions, Thrombotic clot and Embolic clot. Thrombotic clot is most commonly found in atherosclerosis where the fatty acid deposit in the brain arteries leads to blood flow blockage depriving the brain cells of oxygen. Embolic clot or Embolus is a blood clot formed elsewhere in the blood and travels through the blood stream to the brain, thereby causing damage by oxygen deprivation to brain cells. Hemorrhagic stroke is further classified as Intracerebral and subarachnoid hemorrhage. Intracerebral hemorrhage occurs when the blood vessel ruptures and bleeds within the brain. Subarachnoid hemorrhage refers to bleeding in the region between the brain and tissue that surrounds the brain. This phenomenon often occurs in aneurysms (breakage of a weak blood vessel within the wall).

**Figure 4:**
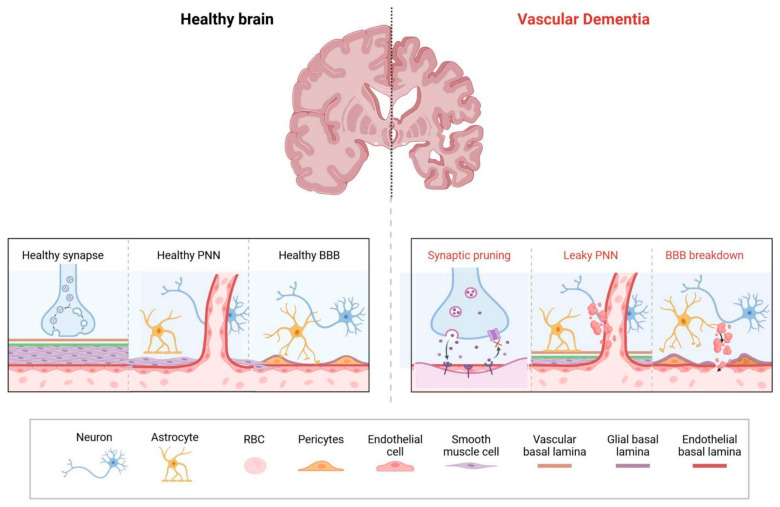
Comparison between a healthy brain and a brain suffering from Vascular Dementia.

There are some genetic risk factors that are associated with buildup and blockages in the blood vessels. Cerebral Autosomal Dominant Arteriopathy with Subcortical Infarcts and Leukoencephalopathy (CADASIL) is a genetic disorder caused by Notch 3 mutations that lead to buildup of material in the blood vessels [26]. Cerebral amyloid angiopathy (CAA) is a condition with genetic risk factors in which amyloid deposits are found to accumulate in the blood vessels of the brain, thereby impeding blood supply. This condition is associated with microbleeds [27]. VaD has also been correlated with the occurrence of Alzheimer’s disease. Up to 50% of instances of dementia cases have reportedly arisen from both vascular problems and Alzheimer’s disease [10,11]. While there have been studies on the genetic factors of VaD, less is known about the changes in the ECM. A review of the literature suggests three main cellular and ultra-structural features of a brain suffering from VaD. Synaptic pruning is hypothesized [28] along with leaky PNNs [20] and breakdown of BBB by MMPs in the diseased brain, and based on our review, we propose a fourth feature, the pathological functionalization and fibrotic remodeling of the fibrillar ECM. A comparative image is shown between the healthy and diseased model of the brain (Figure 4) accounting for the first three; the fourth, pathological functionalization of collagen and fibrotic remolding, is discussed in Section 4.2.

The main features of the brain such as synapse health, robustness of the perineural network and structural integrity of the blood–brain barrier have been highlighted in the images to compare and contrast the changes between the healthy and diseased condition. For the healthy brain, the signaling between the neural synapse and basal lamina of the blood vessel is well regulated, the structural integrity of the perineural network is intact and the blood–brain barrier (BBB) is uniform and robust. In case of the demented brain, the synaptic region is undergoing synaptic pruning and astrogliosis, the perineural network is fragmented and the BBB is damaged, leading to leakage. Created in BioRender. Kalyanasundaram, A. (2026) https://BioRender.com/axqtbny.

## 3. Proteolytic Cascades, Neuroinflammation, and Synaptic Plasticity

### 3.1. Inflammatory Role

Inflammation and its adverse effects appear to be at the core of VaD progression. The inflammatory response associated with VaD has been studied at length, with heavy interest in targeting its players for clinical applications. Failures in blood supply, whether it be by blockage, rupture, or stiffening of the vasculature, are the root of VaD. Disruptions of the blood supply induce oxidative stress as tissues are deprived of oxygen and other nutrients while metabolites from cellular activities are not relieved, which in turn triggers an inflammatory response [3]. Even more, injury of any kind to neuronal tissue, including the vasculature, will also trigger inflammation. Destructive components of this process are heavily involved in the pathogenic changes seen in the neuronal tissue of VaD patients, such as the degradation of the ECM and the reduction in the number of synapses as well as the degeneration of myelin, all of which have implications for memory and cognitive dysfunction [17,29]. Inflammation is also known to weaken the integrity of the blood–brain barrier and hinders proper repair of blood vessels, further exacerbating the impediment of oxygen supply to these tissues [5].

While the causes for VaD pathogenesis seem to be vast, there are broadly identifiable circumstances under which the inflammation of this nature precedes macrocellular tissue damage. Some of these may be genetic, such as in cases of small vessel disease and Type 1 diabetes where impaired or abnormal blood flow is intrinsic to the condition, leading to a chronic and eventually destructive inflammatory response [4]. In other cases, this chronic inflammation is brought on by risk factors often associated with aging, such as hypertension, hardening of the vasculature, cholesterol and plaque buildup in the blood vessels. In all these scenarios, the chronically insufficient blood supply triggers the release of reactive oxygen species and stimulates the production of pro-inflammatory cytokines by the microglia, pericytes, neutrophils and astrocytes, ultimately leading to the activation of various metalloproteinases, which are key to the vascular and neural tissue damage in VaD [5]. There is also crosstalk between inflammatory responses resulting from hypoperfusion in the brain and pathways that promote apoptosis and autophagy, particularly of oligodendrocytes, contributing to white matter damage and cerebral atrophy [30].

Under normal conditions, metabolites of the citric acid cycle and other related oxygen-dependent processes prevent the stabilization of the transcription factor known as hypoxia-inducible factor (HIF). This transcription factor is involved with the upregulation of several cytokines, including IL-1β, IL-6, IL-8, and TNF-α [31]. TNF-α on its own has a direct role in the inhibition of long-term potentiation (LTP) and in apoptosis, particularly in oligodendrocytes and endothelial cells, contributing to both short-term and long-term cognitive impairments as this directly impacts the state of the white matter in the brain. TNF-α is also hypothesized to have a role in the activation of the metalloproteinase system, which will be further discussed below. IL-6 is also known to have negative impacts on long-term potentiation, bringing about the memory deficits that can be seen during and following inflammatory events, while IL-1β has mixed roles in this process, notably in fear conditioning and spatial memory [32]. In addition to heightened levels of these often-damaging cytokines, expression of Brain-Derived Neurotrophic Factor (BDNF) has been found to have long-term reduction in expression levels following a hypoxic event, further compounding the negative cognitive impacts by impairing regular neurogenesis [31,32].

**Figure 5:**
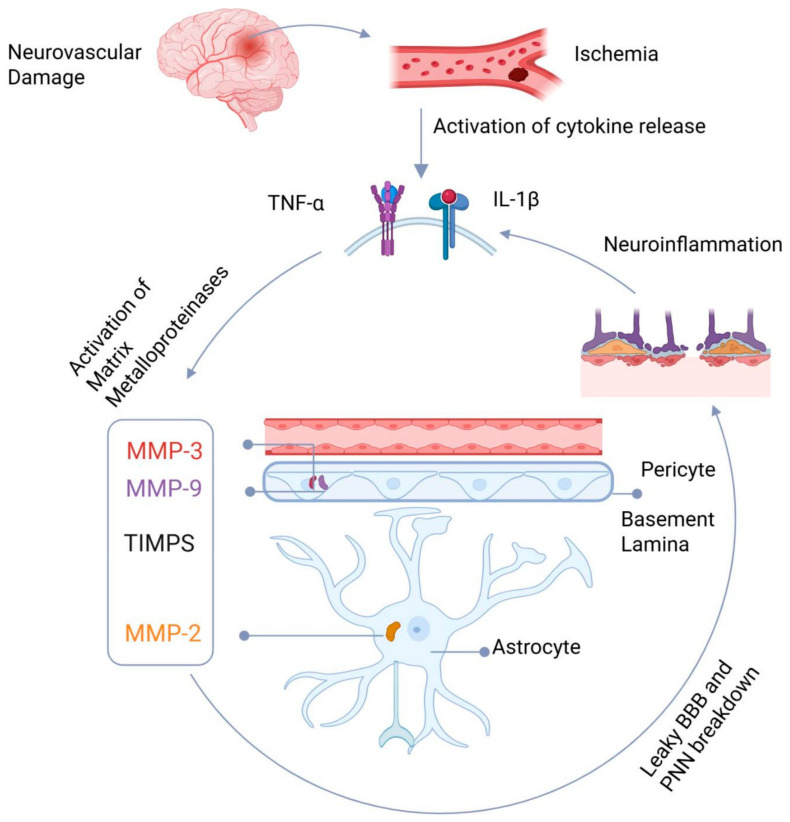
Feedback loop between the inflammation and matrix metalloprotease (MMP) production in blood–brain barrier (BBB). Created in BioRender. Kalyanasundaram, A. (2026) https://BioRender.com/tzikycu.

In cases where an injury has occurred or where the BBB has been compromised in some way, the damage along with the accompanying hypoxia elicits a large and complex immune response. Events such as stroke, hemorrhage, traumatic brain injury and cerebral microbleeds all have characteristic inflammatory responses that involve many of the same pro-inflammatory cytokines. For example, in a study modeling microhemorrhages, microglia were found to migrate to the lesions within minutes where they proliferated for days following. This study also noted the presence of CCR2 and CX3CR1 monocytes at the site of the lesion a few days after their creation, along with increased astrocyte activity [33]. Microglia set the stage for a broader immune response by secreting the pro-inflammatory cytokines IL-1, IL-6, and TNF-ɑ along with reactive oxygen species to recruit other cells of the immune system. In an attempt to repair damaged tissues, the crosstalk between this inflammatory cascade on top of inflammation associated with oxidative stress results in a full-scale reaction that amplifies damage to neural and vascular tissues and gives a likely explanation for the correlation between the frequency and severity of ischemic events and acuity of VaD onset. Following the breakdown of the ECM of the NVU, the resulting protein fragments enter into circulation, which in turn triggers further inflammation [5,20]. A key mechanism seems to be a feedback loop that is activated due to the neuronal damage (Figure 5), which illustrates a simple mechanism where a feedback loop caused during an ischemic condition is highlighted. The neuron damage causes the release of cytokines such as TNF-α and IL-1β. The cytokines activate the production of MMP. MMP 3 and MMP 9 are produced from pericytes, and MMP2 is produced from astrocytes. The release of MMPs leads to proteolytic degradation of the basement membrane and the perineural network, leading to leaky blood–brain barrier and ECM. BBB leakage leads to neuroinflammation to produce TNF-α and IL-1β, hence forming a detrimental feedback loop.

The figure shows an Ischemic condition caused by neurovascular damage such as vascular dementia or traumatic brain injury. This process leads to release of cytokines such as TNF-α and Interleukin 1β. Release of cytokines in turn causes MMP activation in the BBB. The figure shows key MMPs regulating this mechanism—MMP-3 and MMP-9 produced in the pericyte and MMP-2 produced in astrocytes. A stoichiometric balance between these MMPs and their endogenous Tissue Inhibitors of Metalloproteinases (TIMPs) is also normative. Under chronic stress, the depletion or overriding of TIMPs allows uninhibited MMP activity. Un-regulated or maladaptive MMP activity leads to proteolytic degradation of the basement membrane and perineural network, further damaging the BBB. This damage further expedites the production of cytokines, hence causing a feedback loop.

#### MMP/TIMP Stoichiometric Imbalance

The spatiotemporal regulation of matrix metalloproteinases (MMPs), specifically the gelatinases, alongside their endogenous Tissue Inhibitors (TIMPs), serves as a biophysical fulcrum in the maintenance of the blood–brain barrier (BBB) and the homeostatic stability of the neurovascular unit. Under conditions of sustained hemodynamic or hypertensive stress, the local microenvironment is structurally poised to initiate a profound dysregulation of this stoichiometric ratio. Structurally, the non-covalent binding of TIMP molecules, specifically the 28 kDa glycosylated TIMP-1 and the 24 kDa non-glycosylated TIMP-3, to the active catalytic sites of the 72 kDa MMP-2 and 92 kDa MMP-9 occludes the catalytic cleft, thereby arresting matrix degradation through zinc chelation [18].

During vascular pathology and chronic microvascular ischemia, however, this protective balance fails. While TIMP-1 predominantly targets soluble metalloproteinases, TIMP-3 establishes a localized, immobilized inhibitory shield through high-affinity binding directly to the vascular basement membrane. Sustained hypertensive strain leads to a localized depletion or oxidative inactivation of this ECM-bound TIMP-3 reserve. When this localized inhibitor shield is compromised, or when the regional MMP-9/TIMP-1 ratio shifts toward an elevated proteolytic state, the neurovascular unit is subjected to a runaway enzymatic cascade [18]. This stoichiometric shift lowers the net activation energy required to initiate the uncoiling and mechanical yielding of the type IV collagen triple helix, converting a localized hemodynamic stressor into a progressive, neuroinflammatory disruption of the blood–brain barrier.

### 3.2. Clinical Detection Methods

VaD can sometimes be a slow process with different stages of development. In the initial stages it is very mild, and the symptoms are negligible. Hence, it is important to pay attention to small changes in the functional and motor capability of the patients to be able to detect VaD at an early stage.

In clinical settings, even though there are not significant studies available for the role of ECM at each stage of development of VaD, different levels of specific markers that are indicative of an ongoing inflammatory response can be observed across neuroimaging, genetic, and fluid biomarker profiles (Table 3) [34,35]. Two of the MMPs found to be elevated in VaD patients, metalloproteinase-3 and 9, actually tend not to be found in detectable levels unless an insult or a hypoxic event has occurred, further implicating them in the pathogenesis of VaD [17]. This is because in order for these proteases to be found in their active forms, specific cytokines must be present and active to trigger a proteolytic cascade, cleaving the proenzymes for each of these MMPs in tandem. Refer to Figure 5 for the mechanistic overview. Furthermore, there have been studies that have identified microbleeds as an unintended consequence of anti-Aβ immunotherapy, a treatment used to slow the progression of Alzheimer’s disease. This was found along with decreased expression of the inhibitors that prevent the cleavage of the metalloproteinase proenzymes, TIMP1 (Tissue Inhibitor of Metalloproteinases) and TIMP2, which was found to directly increase the incidence of microbleeds, providing additional evidence that these MMPs are a critical destructive force that leads to damage and pathological changes in the ECM and BBB [36]. The exact role of these MMPs in this context is not fully understood, but it is thought that they act either to facilitate the migration of motile immune cells through the BBB or to assist with the breakdown and repair of fibrotic tissue [17,36]. They are known to have a role in the elimination of synapses under regular circumstances.

**Table 3:** The tabular column incorporates information about inflammatory markers and MMPs in vascular dementia.

Biomarker Type	Examples	Relevance to Vascular Dementia	Reference
Neuroimaging	White Matter Hyperintensities (WMHs)	Indicates small vessel disease, chronic ischemia, and cognitive decline	[37]
	Lacunar Infarcts	Small strokes that impair blood flow and lead to cognitive deficits	[4]
	Cerebral Microbleeds	Suggests Col4A1/Col4A2 mutations and fragile blood vessels	[23]
	Brain Atrophy Patterns	Widespread atrophy due to chronic hypoperfusion, different from Alzheimer’s	[35]
Genetic Biomarkers	Col4A1 and Col4A2	Weakens blood vessels, increasing risk of SVD, stroke, and VaD	[38]
	NOTCH3	Causes CADASIL, leading to early-onset vascular dementia	[39]
	APOE-ε4	Increases risk of mixed dementia (VaD + Alzheimer’s)	[40]
Blood Biomarkers	Neurofilament Light Chain (NfL)	Indicates neuronal damage and disease progression	[41,42]
	C-reactive protein (CRP), IL-6	Suggests chronic inflammation, linked to vascular dysfunction	[43]
	Homocysteine	High levels contribute to endothelial damage and stroke risk	[44]
CSF Biomarkers	Amyloid-β and Tau	Helps differentiate VaD from Alzheimer’s	[45]
	Matrix Metalloproteinases (MMP-2, MMP-3, MMP-9)	Involved in blood–brain barrier (BBB) breakdown and vascular impairment; MMP-3 and MMP-9 increase after ischemic events	[17,29,36]
	TIMP1 and TIMP2	Decreased levels reduce MMP inhibition, increasing microbleeds and BBB disruption	[36]
Functional Biomarkers	Executive Function Tests	Detects processing speed and cognitive deficits in VaD	[46]
	Gait Abnormalities	Slowed walking in older adults could signal vascular-related brain damage	[47]

Taken all together, it appears that inflammation in VaD pathogenesis participates in a positive feedback loop that is at the core of the progressive nature of this condition but is seemingly not in and of itself the point of origin. In every case, a pro-inflammatory cascade is triggered by inadequate blood supply or injury with ischemic consequences in an effort to alleviate hypoxia by blood vessel remodeling or to repair injured neural or vascular tissues. The negative downstream effects of this cascade are often detrimental to the integrity of the blood–brain barrier and foster a greater inflammatory response from the damage caused by this attempted remodeling. The extended and amplified inflammatory response and the crosstalk between autophagic and apoptotic pathways result in cerebral atrophy that induces the cognitive deficits that are the face of dementia.

### 3.3. Plasticity

Neuroplasticity is a term used to describe the nervous system’s ability to establish, eliminate and ultimately reorganize synaptic connections, most commonly thought of in the context of learning and healing following injury. Components of the immune response are known to directly interact with and influence synaptic organization, which is at the basis of learning, memory, and overall cognitive functioning [16]. Injuries such as microbleeds, stroke, and hemorrhage are a few of the main risk factors for the development of VaD along with other forms of ischemia due to hardening or buildup in the blood vessels, leading to chronic hypoxia. Each of these conditions results in some kind of inflammatory response, which in cases of both insult and hypoxia persist well after the event occurs. Evidence is also accumulating that cellular activities during periods of reoxygenation are also contributory to neuronal damage [31]. As conditions that lead to VaD tend to be persistent, the chronic and sometimes aggressive inflammation brings about pathological changes in neuronal connectivity [28]. This is then exhibited as cognitive and memory deficits that advance in a progressive manner.

VaD in some ways can be viewed as the pathological outcome of maladaptive or perhaps underequipped responses by neural tissue and the immune system to unfavorable circumstances in terms of blood and oxygen supply. Many of these processes are implicated in normal events and activities in the central nervous system, particularly in neural circuit maintenance and remodeling. In fact, it has even been shown that under mild circumstances, hypoxia induces the expression of both erythropoietin and vascular endothelial growth factor in hippocampal neurons, leading to the differentiation of neuronal precursor cells and increased dendritic spine density, therefore playing an important role in neural plasticity [48]. Despite this apparent necessity for some extent of hypoxia, however, both the hippocampus [31] and deep white matter regions [17] are regarded to be especially vulnerable to hypoxia due to their highly specific oxygen requirements.

In cases of chronic intermittent hypoxia, cellular processes involved with LTP have been implicated in the loss of neuroplasticity. Here, hypoxic conditions promote the release of adenosine by astrocytes as well as the breakdown of extracellular ATP and AMP into adenosine. Extracellular adenosine serves the dual function of inhibiting the activity of voltage-dependent calcium channels in presynaptic neurons and inactivating the glutaminergic N-methyl-D-aspartate receptors (NMDARs) of postsynaptic neurons. Overall, this halts neurotransmission, which in and of itself is a process that accounts for a large portion of neuronal oxygen demand. This mechanism is neuroprotective under benign or mild occurrences of hypoxia, where a temporary decrease in oxygen consumption may prevent further damage. But in cases of more extreme or prolonged hypoxia, these aforementioned processes along with production of reactive oxygen species by microglia resulting in the internalization of ɑ-amino-3-hydroxy-5-methyl-4-isoxazolepropionic acid receptors (AMPARs) create conditions in which glutaminergic signaling is greatly reduced on both presynaptic and postsynaptic ends [31]. The release of glutamate and its interaction with both the NMDARs and the AMPARs is crucial for the fortification of synapses through LTP, which is fundamental to learning and memory formation.

Furthermore, following ischemic conditions, microglia divert from their usual patterns of synaptic surveillance and have been observed making prolonged contact with presynaptic boutons, which oftentimes subsequently leads to their disappearance altogether. This interaction may be promoted by cytokine signaling between the microglia and presynaptic neurons and enhances neurodegeneration [28]. Despite this observation, this study had also found that this loss in synapses was also observed in cases where microglial activity was inhibited. This is corroborated by another study that suggests astrocytes, rather than microglia, are mainly responsible for the pruning of excitatory synapses. By experimentally knocking out the phagocytic receptor of astrocytes, Megf10, the elimination of both presynaptic and postsynaptic terminals of excitatory neurons and therefore the overall turnover of excitatory synapses was greatly reduced in the healthy hippocampus, which in turn disrupted the regular removal of inactive or defective synapses. It was found that the overall effect of this was decreased neuroplasticity and impaired memory formation [49]. This finding opens a new avenue for exploring the interaction between astrocytes and neuronal tissue under hypoxic conditions as the fate of these synapses was also found to be activity dependent.

It has also been suggested that the reoxygenation period following hypoxia may play an even more important role in the cognitive impairment and loss of neural plasticity seen in cases of VaD. During periods of reoxygenation, levels of the inflammatory mediator NFκB have been shown to increase, further exacerbating the inflammatory response even after hypoxic conditions have been resolved. One of the cytokines released in response to hypoxia, IL-1, allows for the phosphorylation of NMDARs through activation of tyrosine kinases. When oxygen supply is replenished, NMDARs are then overwhelmed by a large influx of calcium ions, resulting in a hyperexcitable state that is conducive to neuronal injury. Excessive excitatory signaling along with the inflammatory response that occurs during this period of reoxygenation promote neuronal cell death via apoptosis, ultimately leading to decline in memory and cognitive functioning [31]. This hyperexcitability aspect of intermittent hypoxia provides an interesting possible explanation for the almost tenfold increase in incidence rates of seizures and epilepsy in patients in earlier stages of VaD [50], which could be a contributing factor to the progressive nature of this condition in some patients.

While these inflammatory and excitatory cascades execute the neuronal damage, the primary trigger dictating this cellular fate either directly or with indirect significance, lies within the biophysical architecture of the extracellular matrix itself.

## 4. Molecular Biophysics and Pathological Collagen Functionalization

### 4.1. Extracellular Matrix: The Molecular Structural Architecture of Support and Regulation

The ECM of the CNS is a highly specialized architectural assembly [7,16]. Within the NVU, the ECM is organized into two distinct compartments: the PNNs, which stabilize synaptic connections around neurons, and the basement membrane (BM), a thin, sheet-like scaffold that supports the endothelium and maintains the BBB [8]. The basement membrane is a complex network of Type IV collagen, laminin, fibronectin, nidogens, and heparan sulfate proteoglycans (HSPGs) such as perlecan and agrin. Crucially, the mechanical integrity of the larger cerebral vessels relies on the supramolecular organization of fibrillar collagens (Types I and III) located in the perivascular space [51]. These molecules are not isolated strands but assemble into a crystalline lattice of interdigitating microfibrils organized radially to form fibrils, which are in turn organized into fibril bundles bounded by glycosaminoglycan chains [52,53,54,55,56]. This precise D-periodic architecture provides the tensile strength necessary to withstand hemodynamic pressure and serves as foundational physical defense against vascular rupture. The tensile properties of the blood vessel sheathed in smooth muscle with much stiffer collagen fibrils integrated provide compliance, allowing the vessel to dampen pulsatile flow without triggering an aberrant repair response, to limits imposed by the collagen fibril and fibril bundles [57]. Specifically, healthy cerebral vessels maintain mechanical compliance through the integration of Type III collagen, which inherently possesses a ‘flexi-rod’ molecular architecture. This structure is characterized by flexible amino acid sequences intramolecularly interspersed with rigid, ligand-binding domains [58]. This intrinsic molecular elasticity on the part of Type III collagen, while integrated with stiffer Type I collagen in heterotypic fibrils, buffers hemodynamic strain and protects the NVU from mechanical failure.

Emerging mechanobiological and proteomic data may necessitate a paradigm shift in how we view this collagenous infrastructure. It is not merely a static scaffold but a dynamic, functionalized signaling platform that exists in a spectrum between two distinct states: a ‘Static State’ and a ‘Dynamic State’ [55]. In the healthy, homeostatic brain, the majority of the NVU collagen exists towards the Static State, a quiescent, hydrated scaffold that structurally supports the vessel wall while masking potent bioactive signals, such as the pro-thrombotic GPVI-binding GPO repeats, the inflammatory integrin-binding RGDKGE motifs, and the ‘aborted repair’ HU177 epitope. This organization is tightly maintained by the D-periodic packing of the collagen molecules within their fibril to a highly crystalline degree (including a 67 nm D-periodic repeat with very little variation between individual molecules and a liquid crystalline quasi-hexagonal packing of collagen monomers within these fibrils). Potential cell interaction sites are, by necessity, closely regulated and are hence critical ligand-binding sites within the interior of the fibril that are buried from macromolecular accessibility by closely packed neighboring collagen molecules, bound by ligands such as proteoglycans or shielded beneath the C-terminal telopeptides of surface monomers [55,56]. This gives the fibrillar collagens intelligent fiber properties; the specific organization mediated via specific enzymes and or cells, mechanical circumstances of the tissue, or instances of damage lead to otherwise cryptic cellular and molecular signaling sequences becoming active. Under non-pathological conditions, normal tissue remodeling, growth, development, and repair of damage leads to temporary and regulated exposure of cellularly functionalized collagen fibrils, their normative destination being healthy cell-based interactions, tonal stability and or digestion and replacement with new static (non-functionalized) fibrillar collagen [56].

### 4.2. Pathological Functionalization of the Collagen Fibril

Based upon a significant body of molecular structural data and molecular cell biology derived from other matrix, mechanical, and tissue contexts, a plausible mechanochemical hypothesis for the ultra-structural sequence of events in VaD can be established. In this line of reasoning, the pathology of VaD would initiate when this protective architecture is breached, driving a transition to the Dynamic State at scale and or leading to a cascade of events that reinforce the pathological progression. This transition is not merely degradative or mechanical but architectural at the molecular level, driven by a “mechanochemical” feedback loop involving mechanical stress and enzymatic gating. Chronic hypertension transmits damaging pulsatile energy to the microvasculature, leading to temporal and or permanent mechanical functionalization. High levels of specific molecules, enzymes, and antibodies may take advantage of the temporal exposure of cryptic sites. With higher concentrations, the statistical chance of a negative or unregulated event occurring is increased, while high blood pressure leads to a still greater chance of pathological exposure of these cryptic sites due to mechanically induced decryption and increased accessibility of sensitive bioactive sites.

It is important to distinguish between molecular strain and fibrillar strain in this context. The collagen triple-helix itself is immensely strong, greater than steel of the same diameter, and even the relatively stiffer type I collagen molecule can transition between 10/3 and 7/2 helical conformations to absorb stretch of up to 10% and 20% in laboratory conditions [56,59,60]. However, the fibril bundle typically starts to fail at much lower strains (~3%) due to the rupture of proteoglycan bridges, which will reform as long as hydrated but with an overall reduced total failure stress [61]. Thus, the “functionalization” observed in VaD is not the snapping of the helical peptide backbone. Instead, persistent hypertension likely forces the microfibrils on the exterior of the collagen fibrils into stress-bearing rotations or “untwisting” of the molecular packing of molecules to expose amino acid sequences that are cryptic sites of potential cellular interaction. While the triple-helix “breathes” reversibly, like molecular fluctuations [59,60,62,63], the high-pressure environment forces these surface microfibrils into a rotationally strained state, exposing cryptic sites on Monomers 4 and 5. This is a reversible state (tonal), but if maintained chronically such as due to high blood pressure exerting time averaged, sustained force on the vessel wall, it could lead to functionalization where the cell binding sites may become permanently accessible. If circulating enzymes attack the proteoglycan bridges and or autoimmune antibodies gain access to proteoglycan bridges, they may ‘de-bundle’ the fibril bundle architecture [64], lowering the total tensile tolerance of the vessel wall, and render individual fibrils vulnerable to enzymatic attack, accelerating the failure of the vessel wall in a pathological positive feedback mechanism.

This mechanical vulnerability is modulated by the Vascular Smooth Muscle Cells (VSMCs). The vessel wall is a composite material where VSMCs and collagen operate in parallel. In health, VSMCs actively regulate vascular tone and bear significant load, effectively shielding the collagen scaffold from high strain [57]. However, in VaD and aging, VSMC loss or dedifferentiation transfers the hemodynamic load entirely to the stiff collagen matrix. This unbuffered stress drives the molecular components of the tissue past its yield point(s), triggering further tissue failure and exposure of cryptic sites, cascading the pathology.

**Figure 6:**
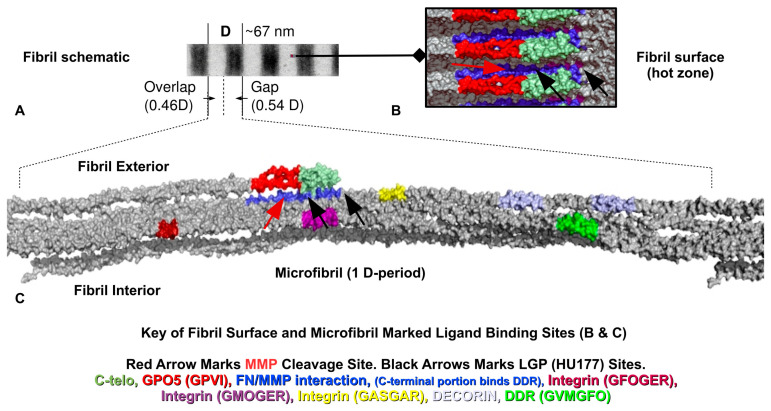
Molecular organization of collagen fibril surface and relative positions of cryptic epitopes and the mechanochemical “hotzone”. (**A**) Fibril schematic: Representation of negative stain fibrillar collagen (type I), dark stain-containing gap region is slightly longer than the light overlap region. For every 5 molecular packing neighbors organized into a microfibril, the 5th molecule only extends part way through the D-period and its C-terminus marks the overlap/gap boundary. A small insert of panel (**B**) is shown and marked with the black line linking panels (**A**) and (**B**), to illustrate relative scale. (**B**) Fibril surface topography: A surface-rendered view (“cell’s eye view”) of the packed molecules of the Type I collagen fibril surface in the cell-interaction domain and specifically the hotzone as defined herein. This spans a zoomed-in region of the fibril surface (the molecular rendering is 2 microfibril assemble layers deep, 3 partial microfibrils wide). The C-terminal region itself contains the GPVI/MHC-factor interaction domains. The coloration highlights the dense convergence of cell-interaction sites clustered around the C-terminal region (red/green), around the buried fibernectin/DDR/MMP interaction domain (blue) with HU177 sites (black arrows). (**C**) Single microfibril: A lateral view of a single microfibril maintaining and expanding the functional coloration from Panel (**B**). Arrows indicate the proposed spatial locations of the HU177 cryptic epitopes, specifically targeting the LPG (Leucine-Proline-Glycine) sequence motifs located directly adjacent to, and strictly on the C-terminal side of, the interstitial collagenase (MMP) cleavage site on Monomer 4 (helical residues 775–776; 3HR2 PDB coordinate file chain A, residues 791–792). The targeted LPG motifs map to helical residues 785–787 and 797–799 (PDB coordinate file chain A, residues 801–803 and 813–815).

We postulate that this mechanical stress renders the collagen susceptible to Enzymatic Gating by MMPs—specifically MMP-2, MMP-3, and MMP-9, which drive the catastrophic failure of the BBB. However, contrary to the view that these enzymes simply “clear the way” or indiscriminately degrade the matrix, collagenolysis is a tightly regulated, structure-dependent process. In the intact fibril, the collagenase cleavage site on Monomer 4 is sterically shielded by the C-telopeptide of Monomer 5 (Figure 6), a “gatekeeper” mechanism [53]. Pathological degradation in VaD, therefore, represents, at least in part, a failure of this architectural defense. We propose that the initial vascular insult (oxidative stress or inflammation) triggers a “priming” event—the cleavage of these protective telopeptides by proteases or reactive oxygen species, mechanical disruption of the fibrils, displacing molecular shielding to allow increased access and/or subsequent cellular adhesions that lead to malformed fibrils and ‘leaky’ blood vessels.

Structural Rationale: While the LPG tripeptide occurs frequently throughout the collagen alpha-chains, the specific motifs identified here are uniquely positioned within a highly metastable cell-interaction “hotzone.” This localized region accommodates the helix-labile MMP cleavage site, the fibronectin-binding domain (which inherently involves local triple-helical unwinding), and the GPVI/MHC-factor interaction regions. Due to the 1D (234-residue) molecular stagger of the fibril, the C-terminal telopeptide of the overlapping adjacent molecule (Monomer 5) physically drapes over the C-terminal side of the cleavage site, masking these specific LPG motifs. The N-terminal approach to the cleavage site lacks this specific displaceable shielding and is devoid of proximate LPG sequences. The convergence of these inherently labile and bioactive domains beneath a single mechanical gatekeeper makes this specific structural locus the prime candidate for the HU177 “aborted repair” signal. The readily unmasked character of this mechanically sensitive zone provides a structural basis for how pathological fibril unbundling triggers a massive, localized inflammatory signaling cascade.

MMP-9, which is elevated in early stroke and cerebral small vessel disease [65,66,67], capitalizes on this compromised state. This gates the fibril, allowing interstitial collagenases (MMP-1, MMP-8) to access the core helix and execute the monomeric shift that fully opens the fibril lattice. Only after this structural “unmasking” can MMP-2 and MMP-9 access the core collagen structure [53]. Once this barrier is breached, MMPs rapidly hydrolyze the basement membrane components (Collagen IV, laminin) and the PNNs (aggrecan, brevican). This degradation compromises the BBB “seal” (mediated by claudin-5 and laminin interactions) and exposes the brain parenchyma to serum proteins, accelerating the neuroinflammatory cascade [17,68]. Concurrently, the structural demolition of these perineuronal nets is heavily driven by the family of A Disintegrin and Metalloproteinase with Thrombospondin Motif (ADAMTS) endopeptidases. Under conditions of ischemic hypoperfusion, the aberrant activation of ADAMTS-4 and ADAMTS-5 selectively cleaves the glutamyl-alanyl or glutamyl-leucyl bonds situated strategically between the highly conserved globular G1 and G2 domains of the aggrecan and brevican core proteins. This precise endopeptidase cleavage untethers the massive polyanionic chondroitin sulfate side chains from their stabilizing hyaluronan backbones, causing a sudden drop in pericellular Donnan osmotic pressure that disrupts local viscoelastic properties and leaves parvalbumin-positive interneurons profoundly vulnerable to excitotoxic injury [12,18,69].

However, the HU177 epitope (LPG), located in Monomer 4 of the alpha-1 chains, lies just C-terminal to the MMP cleavage site and is normally buried directly beneath the folded C-terminal telopeptide of the overlapping Monomer 5 [53,70] (Figure 6). In theory, this unique positioning makes the site highly sensitive to the structural state of the C-telopeptide. Consequently, even without extensive proteolysis, mechanical strain or disruption of the telopeptide gatekeeper would unmask this cryptic site [53,71]. With the exposure of this accessible epitope signal, which sits directly adjacent to the GPVI platelet binding and MHC interaction regions, a state of “aborted repair” is reached, promoting vascular permeability and recruiting inflammatory cells [71,72,73]. This could occur through mechanical damage, an extended period of mechanical strain or frequent pulsations at strain limits of the fibrillar matrix, and of course through rupture and damage with extensive repair events regularly revealing this key epitope.

### 4.3. Bioactive Payload

Glycoprotein VI (GPVI) binding sites (GPO repeats) are exposed on the collagen fibril once the fibril is unmasked of its protective smooth muscle and basement membrane covering. In the “Dynamic” vessel wall, these sites recruit platelets, triggering the release of pro-inflammatory granules and driving “thrombo-inflammation” that further degrades the BBB [74,75,76,77,78]. Crucially, GPVI has also been shown to bind Amyloid-Beta, acting as a nucleation site for cerebral amyloid angiopathy (CAA) [79,80]. Cleavage or mechanical repositioning of the C-telopeptide provides access to HU177 as well as the MMP quarter stagger site and high-affinity cell interaction sites, providing for functionalization of the fibril matrix. Cells will adhere and act, at high concentrations of epitope exposure, which may lead to and or exacerbate an underlying issue. Once the fibril is functionalized, it exposes a “crypticome” of bioactive epitopes that act as ligands for pathological signaling. The HU177 epitope, normally hidden within the molecular packing of Collagen Types I and IV in healthy tissue, becomes exposed and signals a state of aborted repair, promoting vascular permeability and recruiting inflammatory cells [71,72,81]. The unwinding and or unbundling of Type I collagen fibrils also exposes the RGDKGE motif, which binds Integrin αvβ3, activating p38 MAPK pathways that drive endothelial cell proliferation and inflammation, while mechanical strain simultaneously activates parallel ERK 1/2 mechanotransduction pathways in vascular smooth muscle cells [82,83].

The biochemical nomenclature categorizes these fragmented matrix elements based on their structural origin and presentation: they are classified as matricryptins when derived from previously shielded, deeply folded domains of large precursor proteins, and matrikines when explicitly functioning as independent, liberated signaling ligands [22]. For example, the sustained proteolytic cleavage of the non-collagenous NC1 domain of type IV collagen by upregulated MMP-9 generates highly specific matrikines, including arresten (from the α1 chain), canstatin (from the α2 chain), and tumstatin (from the α3 chain). Once exposed or enzymatically unzipped from the stabilizing structural super-lattice, these matrix-derived signaling fragments initiate cascading, receptor-mediated pathways [21].

This proposed mechanochemical sequence both resolves the timeline of VaD progression and serves as a plausible point of origin of neuroinflammation. If accurate, the inflammatory cascade does not spontaneously precede all tissue injury; rather, it may be directly ignited by primary ultra-structural tissue damage, such as the mechanical unwinding and functionalization of the fibrillar matrix. This matrix-driven inflammatory response then acts as an accelerating bridge, ultimately precipitating the severe macro-cellular tissue damage (such as lacunar infarcts, widespread BBB collapse, and neuronal apoptosis) that classically defines the disease.

## 5. Cellular Dynamics, Epidemiological Evidence, and Future Horizons

### 5.1. Cellular Drivers: Fibrotic Transition

The functionalized matrix dictates the fate of perivascular cells, driving a transition from maintenance to fibrosis. Following the initial structural collapse, the NVU attempts to repair the matrix, but in the chronic inflammatory environment of VaD, this process becomes maladaptive. Circulating monocytes are recruited to the perivascular space by matrix signals and, under the influence of the stiff, functionalized matrix and TGF-β, differentiate into Myofibroblasts via the Macrophage-to-Myofibroblast Transition (MMT) [84,85]. These cells are the primary source of the excessive, disorganized Type I collagen that clogs the perivascular space (specifically, congests the Virchow–Robin space). Instead of regenerating the ordered microfibrillar lattice, the vasculature undergoes fibrosis.

Simultaneously, this maladaptive remodeling is propelled from the glial side of the NVU. In response to the hypoxic environment created by initial hypoperfusion, reactive astrocytes undergo altered gene expression, upregulating fibrogenic proteins (laminin, fibronectin) driven by hypoxia-inducible factors (HIF-1) [6,86,87]. Furthermore, the breakdown of the BBB allows plasma Fibrinogen to leak into the Virchow–Robin space, triggering the activation of resident Perivascular Fibroblasts (PVFs) [88,89].

The confluence of these cellular responses, MMT, astrocyte reactivity, and PVF activation results in a fibrotic matrix that is stiffer and far less compliant than the healthy “flexi-rod” architecture [20,90]. This leads to the formation of a rigid vascular scar that impedes glymphatic clearance, exacerbates the initial hypoperfusion, and further drives neurodegeneration [91]. The accumulation of rigid fibrillar collagen restricts the arterial pulsatility needed to drive the convective flow of cerebrospinal fluid (CSF) through the periarterial space. The result is a rigid, dysfunctional vessel wall that fails to support metabolic clearance or synaptic plasticity. Crucially, these structural changes exacerbate the primary drivers of this disease, those being the inability to supply an adequate blood supply by the vasculature and the damaging effects of inflammation, locking the NVU into a cycle of progressive decline and accelerating disease progression.

### 5.2. Clinical Data Supporting the Structural Functionalization Hypothesis

The structural functionalization hypothesis offers a mechanistic basis for observed comorbidities. Patients with Rheumatoid Arthritis (RA) have a significantly increased risk of developing VaD (aHR 1.19), likely due to epitope spreading where circulating RA autoantibodies cross-react with the unwound cerebral collagen fibrils [64,92,93]. Notably, anti-TNF therapy in RA patients reduces this risk [94]. While this non-enzymatic decomposition was initially demonstrated in Type II collagen, the same underlying chemomechanical mechanism was observed in type I fibrillar bundles, which along with type III (and II) share similarities in the way that proteoglycan bands wrap and secure collagen fibrils into larger bundles as fibers. Drawing from models of immune-mediated glomerulonephritis, this implies that circulating RA autoantibodies cross the compromised BBB and cross-react with the functionalized cerebral collagen, converting a localized vascular injury into a potent autoimmune vasculitis [95,96].

Conversely, in the broader context of neurodegeneration, there is a genetically and epidemiologically observed inverse correlation between the incidence of Alzheimer’s disease and Glioblastoma [97,98]. This inverse relationship (RR ~0.53–0.61) is fundamentally driven by shared intracellular pathways and hub genes that dictate cellular fate toward either unchecked proliferation (cancer) or apoptosis (dementia). This suggests a possibility that while both diseases involve the perivascular niche and collagen functionalization, the local immune/matrix “set point” is toggled towards growth (cancer) or degeneration (dementia), driven by inversely regulated matrix modifiers such as MMP-9 [99]. In stark contrast to the oncogenic state, where matrix modifiers relentlessly degrade the ECM to promote hyper-plastic tumor invasion, our proposed mechanochemical model of structural functionalization of VaD suggests that mechanical strain and enzymatic gating drive a maladaptive, fibrotic matrix remodeling. While Alzheimer’s disease and VaD share overlapping end-stage phenotypes, it remains a critical, open research question whether the mechanical and ECM-driven pathologies unique to VaD confer the same anti-oncogenic profile observed in amyloidogenic models.

### 5.3. Limitations and Future Directions

The rigorous characterization of extracellular matrix remodeling in the context of vascular dementia remains constrained by the biophysical limitations inherent in contemporary, static analytical modalities. Current multi-omic methodologies, such as spatial transcriptomics and mass spectrometry-based proteomics, accurately inventory the terminal concentrations of matrix metalloproteinases and cleaved matricryptins. However, these static post-mortem snapshots structurally fail to capture the dynamic, non-linear viscoelastic yielding of the vascular basal lamina under physiological mechanical loads.

There are subject matter boundaries and methodological gaps to bridge with respect to obtaining real-time molecular data of clinical relevance. Although clinical markers are certainly accessible, the instrumentation to record real-time structural ECM clinical data does not yet have the spatial resolution required to see these molecular-scale events. Post-mortem analysis is highly useful because techniques with the necessary resolution can be applied, but then context for clinical relevance has to be extrapolated or correlated. For instance, X-ray diffraction from tissues that are not under dynamic load lacks the temporal resolution required to observe the history-dependent sequential twisting of the fibril and unfolding of the collagen triple-helix under the pulsatile, hemodynamic shear forces characteristic of the living human NVU. Although lacking the accompanying cellular responses to these events, even recording data from such tissues post-loading cycles for comparison with pre-loading would be a significant improvement.

Consequently, testing our hypothesis and, in general, advancing this field in the area of molecular structural considerations will require high-fidelity, dynamic biomechanical modeling coupled with advanced live-cell biology and highly advanced data analytics of clinical observations and epidemiology. In the case of the latter, highly constrained generative AI models have the ability to draw correlations from deeply indirect data, also known as proxy data, with a high degree of statistical rigor [100]. A highly up-to-date approach for constrained deep source assessment in a universal correlation analysis framework was initially applied in quantitative financial signal analysis. Specifically, this Holographic Evaluation System for Topologic Interrelational Analysis (HESTIA) operates as a fundamental mathematical translation layer, mapping the complex topology of systemic failure regardless of the discipline. As such, the framework was designed as a universal correlation analysis system, making use of more than a billion pages of written data to find highly significant relationships using proxy data that lack intuitively clear correlations in more simplistic statistical approaches. Utilizing this, we applied the preliminary model discovery phase of a HESTIA-based analysis regarding possible balance points for accumulating structural changes such as occur in VaD, leading to the hypothesis presented here. This led us to identify peer reviewed literature addressing a causal, statistical balance between VaD and proliferation (see Section 5.2). This initial finding encourages us to pursue this line of research in the future, especially given these very preliminary steps enabled us to both locate key literature confirming this relationship and to map the developing hypothesis against high-fidelity structural data from type I collagen (see Section 4.2 and Section 4.3, and Figure 6).

Another experimentally based future direction involves ex vivo tissue testing in purpose-engineered, closed-loop bioreactor perfusion systems. To accurately recapitulate the pathological biomechanics of cerebral small vessel disease, future experimental models must be capable of subjecting ex vivo vessel constructs to sustained hyper-perfusion velocities and synchronized time-series pressure derivatives that accurately model human systemic hypertension. Dynamic X-ray diffraction of such a system would be an existential practical challenge, but, post-experimental analysis of such stressed tissues might be more viable.

**Figure 7:**
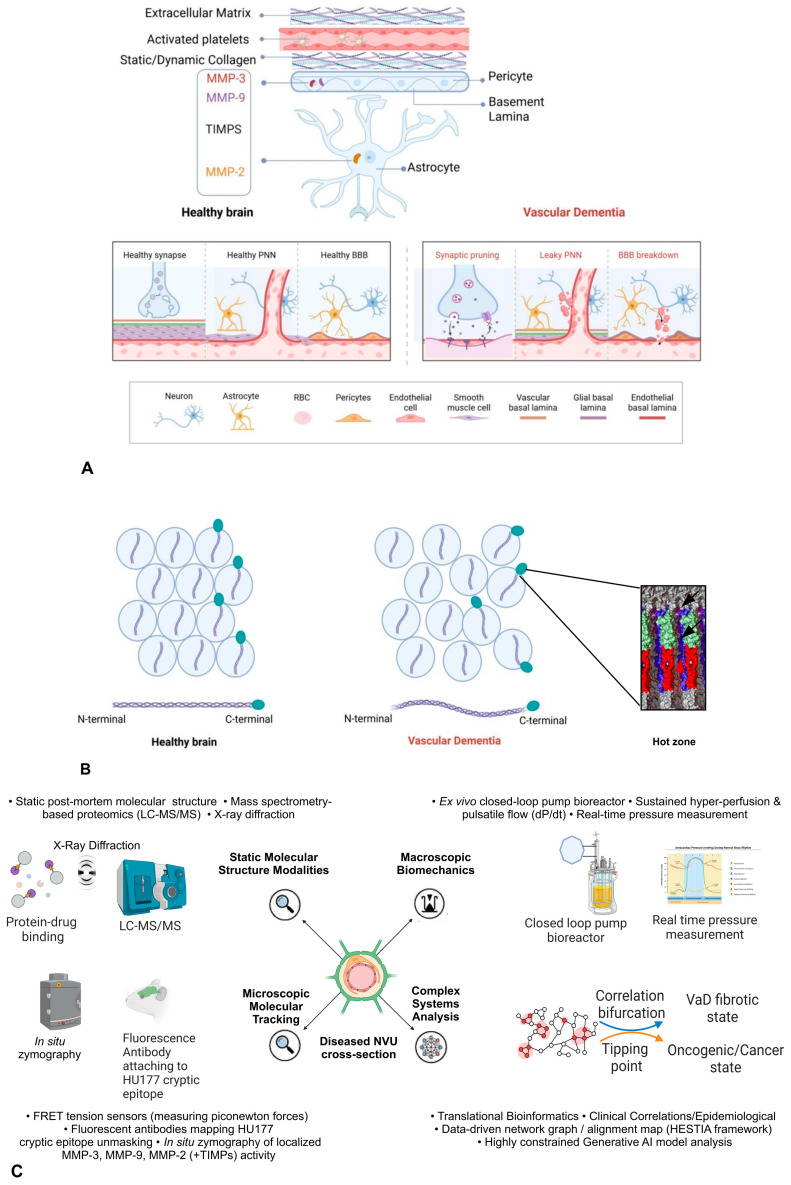
Multiscale schematics of mechanochemical collagen functionalization in VaD and future analytical roadmaps. (**A**) Pathological cascade in the Vascular Dementia (VaD) neurovascular unit (NVU): The schematic contrasts the homeostatic maintenance of a healthy NVU (**left**) with the severe cellular and extracellular matrix (ECM) dysregulation characteristic of VaD (**right**). Chronic hemodynamic strain and subsequent mechanochemical yielding precipitate a downstream cascade driven by localized matrix metalloproteinase (MMP) hyperactivity. BBB breakdown: Degradation of the endothelial and vascular basal laminae compromises blood–brain barrier integrity, leading to red blood cell extravasation and the exposure of thrombogenic ECM components. Crucially, the physical unmasking of collagen allows for Glycoprotein VI (GPVI)-mediated platelet activation. Leaky PNNs: Pathological MMP-3 and MMP-9 activity dismantles the glial basal lamina and perineuronal nets (PNNs), stripping neurons and astrocytic end-feet of their protective structural scaffolding. Synaptic pruning: The ultimate consequence of this continuous matrix demolition is MMP-mediated synaptic stripping and neuronal atrophy, providing a direct mechanistic link between vascular ECM degradation and cognitive decline. (**B**) Molecular-scale mechanochemical yielding of the extracellular matrix: Chronic mechanical strain physically and/or specific targeted structure disruption unbundles fibrils from fibril bundles and can disrupt the collagen super-lattice. Displacement of the protective C-telopeptide “gatekeeper” at the fibril surface unmasks cryptic bioactive epitopes—such as the HU177 motif within the fibrillar “hotzone” (inset; see also Figure 6)—that are otherwise sterically shielded from molecular access such as enzymatic cleavage. The exposure of these vulnerabilities triggers a maladaptive, MMP-mediated inflammatory cascade. As these molecular alterations accumulate within an initially healthy blood vessel, they drive both the continuous degradation of the native matrix and the compensatory, pathological deposition of an abnormal, fibrotic ECM characteristic of VaD. (**C**) Methodological roadmap for identifying ECM pathologies and clinical interventions: Node-based schematic outlines the ongoing integration of physical, molecular, and computational data streams for future VaD characterization and remediation. Each technique listed is intended to be an example representative of the node; the list is non-exhaustive. While Static Molecular Structure Modalities (**top left**) provide the essential baseline data for mapping physical architectures and identifying potential protein–drug binding interfaces, they must be tested under physiological conditions. To achieve this, Macroscopic Biomechanics (**top right**) employs ex vivo closed-loop bioreactors to simulate hypertensive pulsatile flow, while Microscopic Molecular Tracking (**bottom left**) simultaneously visualizes the resulting matrix failure in real-time (e.g., quantifying piconewton mechanotransduction and HU177 unmasking via FRET and zymography). To synthesize diverse multivariable datasets and vast quantities of clinical data, Complex Systems Analysis (**bottom right**) utilizes highly constrained generative AI models (e.g., aHESTIA framework) and translational bioinformatics. This data-driven alignment maps the correlation bifurcation between VaD fibrotic and oncogenic states, ultimately isolating precise targets for novel pharmacological interventions. Selected original elements were incorporated into and the remainder Created in BioRender. Kalyanasundaram, A. (2026) (**A**): https://BioRender.com/1imnogh, (**B**): https://BioRender.com/v91rdy2, (**C**): https://BioRender.com/qwtgm7c.

Parallel to these macroscopic models, advanced molecular techniques could be employed to track the microscopic consequences of mechanical strain. The integration of Förster Resonance Energy Transfer (FRET) tension sensors can map the precise, localized piconewton forces exerted on cellular integrins during lattice deformation. Furthermore, coupling high-resolution multiphoton live-cell imaging with fluorescently labeled antibodies specific to cryptic epitopes (e.g., HU177) will allow for the real-time visualization of mechanochemical unmasking and subsequent inflammatory cell recruitment. Finally, performing time-series multiplexed assays and in situ zymography on the bioreactor effluent will quantify the precise mechanotransduction thresholds required to transition the local MMP/TIMP stoichiometric ratio from homeostatic maintenance to runaway pathological proteolysis. Integrating these dynamic fluid-mechanics platforms with high-resolution cellular tracking will be vital for mapping extracellular matrix demolition as a continuous, flow-dependent variable, providing a rigorous foundation for future therapeutic interventions. Figure 7 summarizes this multiscale mechanochemical methodological and analytical future direction, alongside a recap of the developing pathological functionalization of the NVU presented here.

### 5.4. Conclusion: A Collagen Code of Dementia?

VaD is thus potentially a disease of pathological Collagen Functionalization. The ECM in the NVU of the brain is not only a structural support but acts as a key factor in cell signaling and thus during disease pathogenesis. It is a significant factor in initiation, not just in remodeling. The transition of the NVU matrix from a protective Static State to a reactive Dynamic State unmasks bioactive epitopes, HU177, RGDKGE, and exposed GPO, that recruit the immune system and direct the formation of a fibrotic scar. The active role that the ECM plays in VaD pathogenesis, progression, and presentation is complex and has yet to be fully understood. However, it appears that the maladaptive changes in the neuronal and vascular ECM composition brought about by proteolytic activity, mechanical strain, and alterations in protein expression induce a dysregulated state that contributes immensely to neuronal atrophy and dysfunction. To better explain features of this disorder, such as targeted cell loss, it may be beneficial to further characterize the cell-to-matrix interactions that occur in neuronal tissue. By studying how the ECM and its associated cellular network influence each other, a greater understanding could be gained about how neurological dysfunctions arise in the first place and possibly pose a link between seemingly unrelated disease-states, such as epilepsy and dementia. Future therapeutic strategies could move beyond hemodynamics to target this structural transition. For instance, by utilizing monoclonal antibodies targeted strictly against these unmasked cryptic epitopes (such as HU177), or by engineering pharmacological agents that stabilize the collagen fibril bundles and reinforce the native gatekeeper mechanism, it may be possible to halt the inflammatory cascade at its origin. In addition, this knowledge could also shed light on the mechanisms underlying different presentations of VaD, potentially leading to more individualized treatments. This, along with the advancing understanding of immunological interplay, is crucial for the mitigation of and improving therapeutics for VaD, along with many other neurological conditions.

## Figures and Tables

**Table 1:** Proposed conceptual framework for the spatiotemporal progression of NVU pathology in VaD (see also Figure 2).

Category	Stage 1: Cognitive Impairment	Stage 2: Early Stages	Stage 3: Middle Stages	Stage 4: Late Stages	References
Memory Impairments	Forgetfulness and misplaced items	Repeating memories, inability to recall names	Unable to recognize familiar people or places	No short-term memory, inability to form new memories	[1,2]
Speech Impairments	–	Slowed speech	Substituting words, Incomplete sentences	Gibberish	[13]
Physical Impairments	Gait impairment, poor coordination	Parkinson, poor coordination	Impaired posture, gait and movement, sleep apnea	Incontinence, sleep apnea	[4,10]
Behavioral Instability	Depression, lack of personal care, hoarding	Wandering, delusion/paranoia, confusion	wandering, anxiety, hallucination, paranoia	Wandering	[10]
Emotional Instability	Agitation, mood swings	Anxiety, mood swings	Apathy, aggression	Aggression, mood swings	[1]

## Data Availability

No new data were created or analyzed in this study. Data sharing is not applicable to this article.
